# Mismatch repair markers in preoperative and operative endometrial cancer samples; expression concordance and prognostic value

**DOI:** 10.1038/s41416-022-02063-3

**Published:** 2022-12-08

**Authors:** Hege F. Berg, Hilde Engerud, Madeleine Myrvold, Hilde E. Lien, Marta Espevold Hjelmeland, Mari K. Halle, Kathrine Woie, Erling A. Hoivik, Ingfrid S. Haldorsen, Olav Vintermyr, Jone Trovik, Camilla Krakstad

**Affiliations:** 1 Centre for Cancer Biomarkers CCBIO, Department of Clinical Science, University of Bergen, Bergen, Norway; 2 Department of Obstetrics and Gynecology, Haukeland University Hospital, Bergen, Norway; 3 Section of Radiology, Department of Clinical Medicine, University of Bergen, Bergen, Norway; 4 Mohn Medical Imaging and Visualization Centre, Department of Radiology, Haukeland University Hospital, Bergen, Norway; 5 Department of Pathology, Haukeland University Hospital, Bergen, Norway; 6 Gade Laboratory for Pathology, Department of Clinical Medicine, University of Bergen, Bergen, Norway

**Keywords:** Endometrial cancer, DNA mismatch repair, Endometrial cancer, Prognostic markers, Gynaecological cancer

## Abstract

**Background:**

The endometrial cancer mismatch repair (MMR) deficient subgroup is defined by loss of MSH6, MSH2, PMS2 or MLH1. We compare MMR status in paired preoperative and operative samples and investigate the prognostic impact of differential MMR protein expression levels.

**Methods:**

Tumour lesions from 1058 endometrial cancer patients were immunohistochemically stained for MSH6, MSH2, PMS2 and MLH1. MMR protein expression was evaluated as loss or intact to determine MMR status, or by staining index to evaluate the prognostic potential of differential expression. Gene expression data from a local (*n* = 235) and the TCGA (*n* = 524) endometrial cancer cohorts was used for validation.

**Results:**

We identified a substantial agreement in MMR status between paired curettage and hysterectomy samples. Individual high expression of all four MMR markers associated with non-endometrioid subtype, and high MSH6 or MSH2 strongly associated with several aggressive disease characteristics including high tumour grade and FIGO stage, and for MSH6, with lymph node metastasis. In multivariate Cox analysis, MSH6 remained an independent prognostic marker, also within the endometrioid low-grade subgroup (*P* < 0.001).

**Conclusion:**

We demonstrate that in addition to determine MMR status, MMR protein expression levels, particularly MSH6, may add prognostic information in endometrial cancer.

## Introduction

Endometrial cancer is the most common gynaecological cancer in countries with high human developmental index and the incidence of endometrial cancer is increasing [1]. Preoperative risk stratification of patients relies largely on the assessment of tumour histology with the addition of imaging in many hospitals and is reliable but not optimal for all patients [2]. Inaccurate risk allocation may result in both over- and under-treatment of patients, associated with more side effects or a higher risk of recurrence as outcome. Improved methods for patient stratification are therefore needed. Molecular subclassification of endometrial tumours, as defined by The Cancer Genome Atlas (TCGA) [3], improves prognostication and is included in the recent ESGO/ESTRO/ESP guidelines for the management of endometrial cancer [4]. These subtypes include the POLE ultramutated, microsatellite instability (MSI) hypermutated, copy number high (or p53 abnormal) and copy number low (or no specific molecular profile).

Patients with MSI-hypermutated tumours have an intermediate prognosis [5] and may be eligible for treatment with immune checkpoint inhibitors [6], although this is seldom used in clinical routine. The MSI-hypermutated subgroup can be detected by immunohistochemical (IHC) staining of the DNA mismatch repair (MMR) markers MSH6, MSH2, PMS2 and MLH1, where the loss of any of these defines MMR deficiency (MMR-D), a surrogate marker for MSI. MSH2 and MSH6 form the heterodimer MutSα, whereas PMS2 and MLH1 dimerise to form MutLα [7, 8]. Both dimers are key players in MMR-associated genome maintenance. During normal DNA replication and recombination, the MMR system recognises and corrects base–base mismatches and insertion/deletion mispairs. MMR can also prevent homologous recombination [8]. Deficient MMR reduces the ability to identify errors during DNA replication and results in MSI [9, 10], a hypermutated phenotype, genomic instability and resistance to alkylating chemotherapeutic agents [8].

Following the new guidelines for endometrial cancer, detection of MMR-D/MSI in preoperative biopsy is entering clinical workup. The most common methods for detection are IHC staining of the MMR proteins to define MMR status or PCR-based methods to define microsatellite stability (MSI versus MSS). IHC is frequently used and recommended for endometrial cancer [4, 11, 12], as this is a reliable method with low cost, provides information on the altered gene/protein and is easily performed using small preoperative formalin-fixed biopsies. However, concordance in expression pattern between preoperative and operative biopsies should be determined. In addition, when implementing use in the clinic, the potential prognostic value of the markers should also be investigated. In prostate cancer, overexpression of MSH6, MLH1 and PMS2 proteins associates with poor disease outcome and genetic instability in a cohort of 11,152 prostate cancer patients [13]. Overexpression of MMR proteins is also linked to poor survival in oral squamous carcinoma (MSH2, MSH6) [14], bladder cancer (MLH1) [15], Stage I–II colon cancer (MLH1) [16] and Stage I–III melanoma (MSH6) [17]. In contrast, increased mRNA expression of *PMS2* is associated with improved overall survival in ovarian cancer [18], MSH2 fails to predict progression-free survival in bladder cancer and high MSH2 predicts improved overall survival in Stage I–II colon cancer [16]. To our knowledge, only one study has explored differential MMR protein expression in relation to prognosis in endometrial cancer, where high MSH6 (RNA and protein) associated with poor disease-free survival in a cohort of 243 patients with mostly endometrioid histologies [19].

Here, we aim to evaluate the concordance of MMR status in preoperative and operative samples and explore the prognostic value of MMR protein expression in a large prospectively collected population-based endometrial cancer cohort.

## Materials and methods

### Patient series

A population-based patient series have been prospectively collected at Haukeland University Hospital, Norway from 2001–2015 and includes both curettage and hysterectomy biopsies from women diagnosed with endometrial cancer. In total, 1694 lesions from 1058 patients, including preoperative curettage (*n* = 761) and hysterectomy (*n* = 933) specimens, were included in the present study. Patients were surgically staged according to the International Federation of Gynecology and Obstetrics (FIGO) 2009 criteria [20]. Clinical data were collected as previously described [21]. When available, fresh frozen tissue was collected in parallel with formalin-fixed paraffin-embedded (FFPE) tissue and used for mRNA extraction. Hormone receptor status from curettage specimens was available from a previous study [22].

### Immunohistochemistry

Tissue microarrays (TMAs) were constructed from FFPE curettage and hysterectomy biopsies as previously described [23, 24]. The TMA slides (5 µm) were dewaxed in xylene and rehydrated in ethanol before antigen retrieval in target retrieval solution at pH 9 or pH 6 for 15 min in microwave followed by peroxidase block (S2023, Dako, Glostrup, Denmark) for 8 min at room temperature. Sections were incubated for 60 min at room temperature with anti-MSH6 monoclonal mouse antibody (1:25, NCL-L-MSH6, NovocastraTM, Leica Biosystems Newcastle, United Kingdom) or anti-PMS2 monoclonal mouse antibody (1:25, PMS2-L-CE; NovocastraTM, Leica Biosystems Wetzlar, Germany) or incubated for 30 min at room temperature with anti-MLH1 monoclonal mouse antibody (1:100; NCL-L-MLH1; NovocastraTM, Leica Biosystems Newcastle, UK) or anti-MSH2 monoclonal mouse antibody (1:50; NCL-l-MSH2–612; NovocastraTM, Leica Biosystems Newcastle, UK). All sections were incubated for 30 min with a secondary anti-mouse antibody (Agilent Technologies, Santa Clara, USA). Staining was visualised with diaminobenzidine peroxidase (DAB) (EnVision detection system, K3468, Dako, Glostrup, Denmark). Sections were counterstained with hematoxylin (S3301, Dako, Glostrup, Denmark) before dehydration and mounting.

### Evaluation of staining

To determine MMR status, sections were scored visually for loss or intact expression of the MMR proteins: MSH6, MSH2, MLH1 and PMS2. MMR-D was defined as the complete or subclonal loss of one or more MMR proteins [25, 26]. Full sections were used in cases with a lack of positive internal stromal control, and cases were excluded from the study if both the TMA cores and the full section lacked stromal expression (*n* = 16 patients in curettage series, *n* = 49 patients in hysterectomy series).

For the evaluation of individual MMR proteins as prognostic markers, protein expression was evaluated using the semi-quantitative staining index (SI) method. SI was calculated as a product of intensity (0 = negative, 1 = weak, 2 = moderate, 3 = strong) and area (0 = 0%, 1 = less than 10%, 2 = 10–50%, 3 = more than 50% of tissue had positive staining). Evaluation of staining was performed blinded for clinical characteristics and outcome. In statistical analyses, a Youden index was performed to identify best prognostic cut-offs. For MSH6 and MSH2, the low expression group included samples with SI 0, 1, 2, 3 and 4 and the high expression group with SI 6 and 9 both in curettage and hysterectomy specimens. Same cutoff was used for PMS2 in curettage samples, whereas in hysterectomy samples, the high expression group included SI 4, 6 and 9. For MLH1, the high expression group included index 9 in both hysterectomy and curettage specimens. A subset of ~100 samples was scored by two independent observers and Kappa values were calculated to investigate inter-observer reproducibility. Kappa values in curettage were 0.78, 0.75, 0.86 and 0.71 and in hysterectomy 0.84, 0.68, 0.61 and 0.67 for MSH6, PMS2, MLH1 and MLH2, respectively.

### MSI assay

Tumour DNA from snap frozen hysterectomy tissues (*n* = 60 patients) was extracted using AllPrep DNA/RNA mini kit (QIAGEN). DNA was analysed by multiplex PCR using the five mononucleotide repeat markers, *NR-21*, *NR-24*, *MONO-27*, *BAT25* and *BAT26* (MD1641, Promega Corporation, Madison, WI, USA). Tumours with two or more markers that were positive for shifts in the allelic bands were classified as MSI, whereas tumours with one or without unstable marker were classified as MSI-L and MSS, respectively. MSI-L was considered MSS in subsequent analysis.

### Transcriptomic datasets

mRNA expression data of *MLH1, MSH2, MSH6* and *PMS2* was available from a subset of 256 patients [27]. When more than one probe was available for one gene, max probe expression was chosen. The Cancer Genome Atlas (TCGA) transcriptomic dataset (Uterine Corpus Carcinoma, PanCancer Atlas, *n* = 524) [28] was downloaded from the cBioPortal: https://www.cbioportal.org/datasets.

### Statistical analysis

Statistical analyses were performed using IBM SPSS Statistics for Macintosh (version 25.0, IBM Corp., Armonk, NY, USA) or RStudio (version 1.4., RStudio, Boston, MA, USA). A level of significance was set at *P* < 0.05 and all *P* values were two-sided. Agreement between two methods or tissue types were evaluated by Cohen’s Kappa statistics. Pearson Chi-square test and Mann–Whitney *U* test was used for comparison between groups of categorical and continuous variables, respectively. Survival analyses for disease-specific survival (DSS) were generated using the Kaplan–Meier method, and differences between groups were compared using the log-rank (Mantel–Cox) test. The entry date was defined as the time of primary surgery. Endpoint was defined as the time of death due to endometrial cancer. Patients who died from other causes or were lost to follow-up were censored. Cox proportional hazard modelling was used to estimate the effect of covariates on the hazard rate. The proportional hazard assumption was evaluated by a graphical assessment of risk factors over time. Due to the high correlation between oestrogen receptor (ER) and progesterone receptor (PR) expression, these two variables were combined into one covariate. Low ER/PR expression includes loss/low expression of both receptors, and high includes the remaining combinations (low/high, high/low, high/high).

### Reporting summary

Further information on experimental design is available in the Nature Research Reporting Summary linked to this paper.

## Results

### Substantial agreement in MMR status between preoperative curettage and hysterectomy specimens

**Fig. 1:**
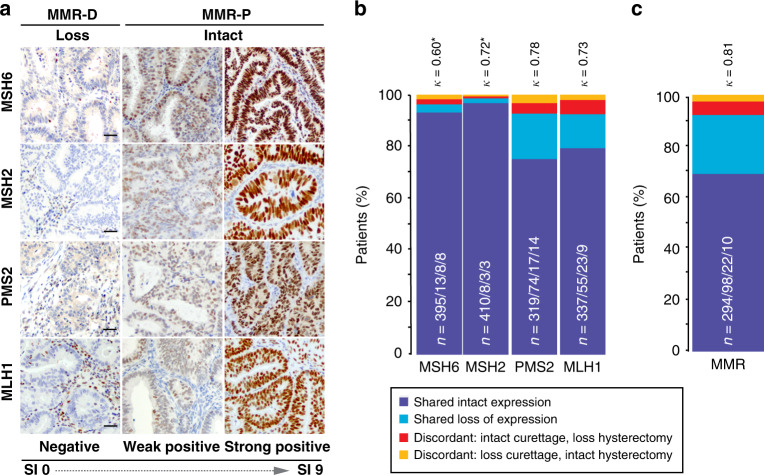
Concordant MMR protein expression between paired curettage and hysterectomy samples (*n* = 424). Mismatch repair (MMR) deficiency was defined as loss of one or more MMR proteins as indicated above images. Differential expression levels were defined by staining index (SI) 0–9 (0, 1, 2, 3, 4, 6 and 9), as indicated below images (**a**). A moderate agreement in MSH6 expression and a substantial agreement in MSH2, PMS2 and MLH1 expression was found between pairs of curettage- and hysterectomy samples (**b**). Paired samples showed substantial concordance in MMR status (MMR-D versus MMR-P) (**c**). κ Cohen’s kappa coefficient, MSH6 MutS Homolog 6, MSH2 MutS Homolog 2, PMS2 PMS2 Homolog 2, MLH1 MutL Homolog 1, MMR-D mismatch repair deficient, MMR-P mismatch repair proficient. *Mean prevalence of MSH6 and MSH2 loss is <10% implying that κ values are conservative estimates. N = Shared intact expression/shared loss of expression/discordant: intact curettage, loss hysterectomy/discordant: loss curettage, intact hysterectomy.

Tumours were considered MMR-D if one or more MMR protein(s) showed loss of expression (Fig. 1a). When considering all available samples with status on all four MMR proteins, loss of MSH6 or MSH2 was detected in few curettages (loss in 5.3% or 2.4%, respectively) and hysterectomy (loss in 5.2% or 2.4%, respectively) samples. PMS2 or MLH1 expression was lost in a higher fraction of the tumours, both in curettage (20.6% or 15.6%, respectively) and in hysterectomy samples (21.3% or 17.6%, respectively) (Table 1). Biomarkers that inform primary treatment are usually investigated in the preoperative sample and seldom re-evaluated in the operative sample. However, molecular class reported in research is often defined from operative samples [5, 11, 29, 30]. Paired samples from a subset of 424 patients were investigated to evaluate the consistency in defined MMR status. MSH2, PMS2 and MLH1 showed substantial agreement between samples with Cohen’s κ of 0.72, 0.78 and 0.73 (*P* < 0.001), respectively, while MSH6 expression showed moderate agreement with a Cohen’s κ of 0.60 (*P* < 0.001) (Fig. 1b). Notably, of the discordant PMS2 and MLH1 cases, MMR status was more frequently detected as intact in curettage and lost in hysterectomy, than lost in curettage and intact in hysterectomy (Fig. 1b). Thus, PMS2 and MLH1 were more frequently detected as loss after re-evaluation of the hysterectomy sample.

**Table 1:** MMR protein loss/intact expression detected at similar frequencies in curettage and hysterectomy series.

	MutSα				MutLα			
	MSH6		MSH2		PMS2		MLH1	
	Intact (%)	Loss (%)	Intact (%)	Loss (%)	Intact (%)	Loss (%)	Intact (%)	Loss (%)
Curettage (*n* = 582)	550 (94.7)	31 (5.3)	567 (97.5)	14 (2.4)	450 (79.4)	117 (20.6)	491 (84.4)	91 (15.6)
Hysterectomy (*n* = 753)	709 (94.8)	39 (5.2)	730 (97.6)	18 (2.4)	566 (78.7)	153 (21.3)	609 (82.4)	130 (17.6)

Missing information on curettage due to negative stromal straining of MSH6 and MSH2 for one patient and PMS2 for 15 patients. Data missing on hysterectomy due to negative stromal straining of MSH6 and MSH2 for five patients, PMS2 for 24 patients, MLH1 14 patients.

MMR status was determined in both preoperative and operative patient series. For the full cohort, MMR-D was detected in 25.8% of preoperative curettage samples and 28.0% of hysterectomy samples, based on the loss of one or more MMR proteins. For a subset of 60 operative samples, MSS/MSI status was available from PCR assay. A high level of concordance was seen between the two methods, with Cohen’s κ = 0.80 (*P* < 0.001). To assess whether preoperative MMR status captures all patients with MMR-D tumours, we compared MMR status from the curettage sample with MMR status from the hysterectomy sample (Fig. 1c). In this paired sample set, MMR-D was detected in 25.8% of preoperative samples and 28.3% of operative samples. Concordant MMR status between sample pairs was found in 392/424 (92.4%) patients. Discordant sample pairs were observed in 32/424 (7.6%) patients, of which 10/424 (2.7%) were MMR-D in preoperative samples and MMR-proficient (MMR-P) in operative samples and 22/424 (5.2%) were MMR-P in preoperative samples and MMR-D in operative samples (Cohen’s κ: 0.81, *P* < 0.001). As expected, MMR status was not prognostic with a 5-year DSS of 82% for preoperative MMR-D and 83% for MMR-P patients (*P* = 0.913) (Supplementary Fig. 1).

### High expression of MSH6, MSH2 or PMS2 in preoperative curettage associates with aggressive disease and poor outcome

To determine if a more thorough evaluation of MMR protein expression may add relevant information to the diagnostic workup for endometrial cancer, we evaluated the differential expression of MSH6, MSH2, PMS2 and MLH1 using staining index (Fig. 1a) in both curettage and hysterectomy specimens. High MSH6 or MSH2 strongly associated with aggressive tumour characteristics, including high-grade endometrioid and non-endometrioid histological types, advanced FIGO stage, aneuploidy, as well as lymph node metastasis for high MSH6 (*P* < 0.05 for all) (Table 2). High PMS2 and MLH1 significantly associated with non-endometrioid subtype (*P* < 0.001) but did not significantly associate with other clinicopathological features. Similar findings were observed for MMR protein expression in hysterectomy specimens (Supplementary Table 1), with the strongest association between high MSH6 and aggressive disease features.

**Table 2:** MMR protein expression associates with clinicopathological variables in curettage specimens.

	MSH6			MSH2			PMS2			MLH1		
Variable	Low, *n* (%)	High, *n* (%)	*P* value	Low, *n* (%)	High, *n* (%)	*P* value	Low, *n* (%)	High, *n* (%)	*P* value	Low, *n* (%)	High, *n* (%)	*P* value
Number of patients	302	239		280	386		287	230		204	455	
Age			**0.013**			0.141			**0.019**			0.494
<66	169 (61)	108 (39)		154 (45)	190 (55)		167 (60)	110 (40)		109 (32)	230 (68)	
≥66	133 (50)	131 (50)		126 (39)	196 (61)		120 (50)	120 (50)		95 (30)	225 (70)	
Histologic type*			**<0.001**			**0.036**			**<0.001**			**<0.001**
Endometrioid	273 (61)	177 (39)		241 (44)	308 (56)		255 (61)	162 (39)		191 (35)	350 (65)	
Non-endometrioid	29 (32)	62 (68)		39 (33)	78 (67)		32 (32)	68 (68)		13 (11)	105 (89)	
Clear cell	7 (50)	7 (50)		11 (48)	12 (52)		5 (29)	12 (71)		5 (24)	16 (76)	
Serous papillary	16 (33)	33 (67)		19 (32)	40 (68)		15 (30)	35 (70)		5 (8)	56 (92)	
Carcinosarcoma	2 (10)	18 (90)		7 (27)	19 (73)		9 (37)	15 (63)		2 (7)	56 (93)	
Undifferentiated/other	4 (50)	4 (50)		2 (22)	7 (78)		3 (33)	6 (67)		1 (12)	7 (88)	
Histologic grade**			**<0.001**			**0.021**			0.293			0.855
Grade 1–2	242 (64)	135 (36)		213 (46)	249 (54)		222 (63)	132 (37)		163 (36)	289 (64)	
Grade 3	28 (42)	39 (58)		25 (32)	53 (68)		31 (55)	25 (45)		28 (35)	52 (65)	
Myometrial infiltration			**0.016**			0.250			0.610			0.331
<50%	197 (60)	129 (40)		179 (45)	222 (55)		184 (58)	136 (42)		118 (30)	277 (70)	
≥50%	91 (49)	93 (51)		91 (40)	137 (60)		92 (55)	75 (45)		76 (34)	150 (66)	
FIGO stage			**<0.001**			**0.004**			0.224			0.906
I–II	271 (59)	187 (41)		250 (45)	312 (55)		248 (57)	189 (43)		170 (31)	380 (69)	
III–IV	30 (37)	52 (63)		30 (29)	73 (71)		39 (49)	40 (51)		34 (32)	74 (68)	
Ploidy			**<0.001**			**0.014**			0.080			0.053
Diploid	154 (60)	102 (40)		135 (45)	163 (55)		141 (62)	88 (38)		109 (37)	186 (63)	
Aneuploid	16 (26)	45 (74)		23 (30)	54 (70)		30 (49)	31 (51)		20 (25)	59 (75)	
Metastatic lymph nodes			**0.008**			0.207			0.742			0.188
Negative	213 (58)	151 (42)		196 (45)	240 (55)		190 (56)	148 (44)		130 (30)	303 (70)	
Positive	17 (38)	28 (62)		19 (36)	34 (64)		23 (59)	16 (41)		22 (39)	35 (61)	

*FIGO* International Federation of Gynaecologist and Obstetrics, *n* number of patients, *MSH6* MutS homolog 6, *MSH2* MutS homolog 2, *PMS2* PMS1 homolog 2, *MLH1* MutL homolog 1.

Low: SI 0–4, high: SI 6–9.

Statistically significant *P*-values (<0.05) are bold.

*Comparing endometrioid vs. non-endometrioid.

**Only endometrioid histology.

Data missing on histologic grade for 6 (MSH6), 9 (MSH2, MLH1), and 7 (PMS2) patients, myometrial infiltration for 31 (MSH6), 37 (MSH2), 30 (PMS2) and 38 (MLH1) patients, FIGO classification for 1 patient (MSH6, MSH2, PMS2, MLH1), ploidy for 224 (MSH6), 291 (MSH2), 227 (PMS2), 285 (MLH1) patients, metastatic lymph nodes for 132 (MSH6), 177 (MSH2), 140 (PMS2) and 126 (MLH1) patients.

**Fig. 2:**
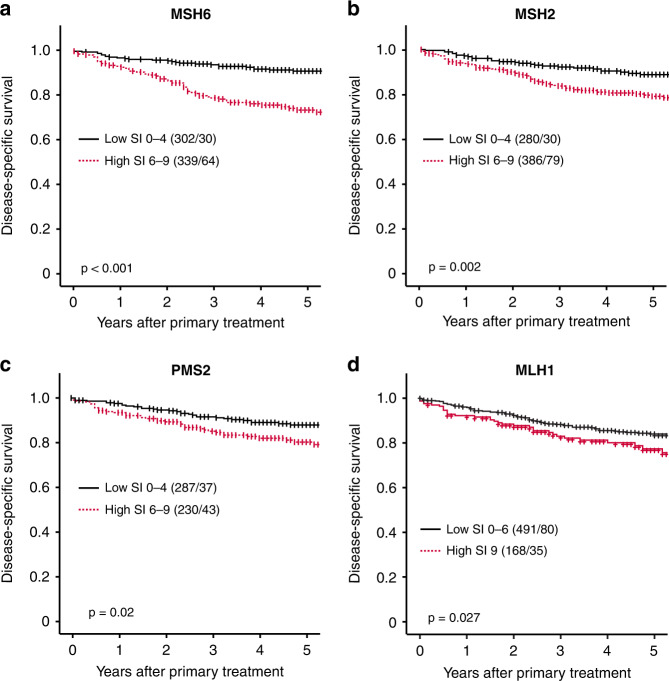
Expression of MSH6, MSH2, PMS2 and MLH1 is prognostic in preoperative lesions. High individual expression of MSH6, MSH2, PMS2 and MLH1 in curettage was associated with poor disease-specific survival (**a**–**d**). Cut-offs determined by Youden Index. *P* values are given by log-rank (Mantel–Cox) analysis. Number in brackets indicates the total number of patients/number of events. Abbreviations: MutS Homolog 6 (MSH6), MutS Homolog 2 (MSH2), PMS2 Homolog 2 (PMS2), MutL Homolog 1 (MLH1).

In curettages, individual high MSH6, MSH2, PMS2 and MLH1 significantly associated with poor prognosis, with a 5-year DSS of 73%, 79%, 81% and 79%, compared to 90%, 88%, 87% and 84% for low expression, respectively (*P* ≤ 0.03 for all; Fig. 2). Except for MLH1, these findings were validated in hysterectomy specimens (Supplementary Fig. 2). In a subset of hysterectomy samples with available transcriptomic data (*n* = 256), protein expression significantly correlated with mRNA expression levels (*P* = 0.029, 0.003 and <0.001, respectively) for MSH6, MSH2 and MLH1 but not for PMS2 (Supplementary Fig. 3A). High mRNA expression of *MSH6*, *MSH2* and *PMS2* significantly associated with poor 5-year DSS (*P* ≤ 0.02 for all; Supplementary Fig. 3B).

### MSH6 is an independent preoperative prognostic marker, also in low-grade endometrial cancer

**Fig. 3:**
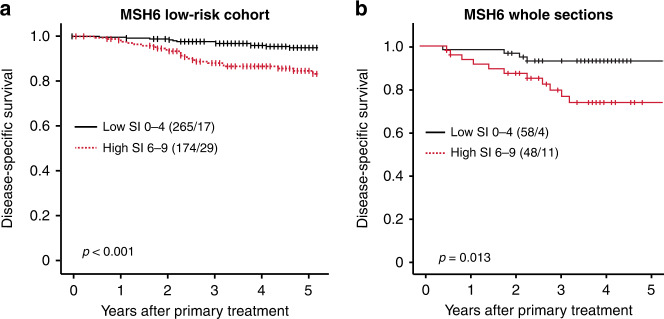
MSH6 predicts poor outcome within the low-grade endometrioid subgroup and validates as a prognostic marker in whole curettage sections. High MSH6 predicts poor prognosis in preoperative low-grade endometrioid tumours (**a**). Evaluating MSH6 expression using whole sections validates the prognostic effect (**b**). MSH6 MutS Homolog 6.

The independent prognostic value of MSH6, MSH2 or PMS2 was evaluated using multivariate Cox analysis, after adjusting for variables currently in use for preoperative risk stratification. Contrasting MSH2 and PMS2, MSH6 demonstrated independent prognostic impact in curettage samples, when adjusting for age, curettage histology and hormone receptor status, with a multivariate HR of 2.8 (95% CI 1.5–5.3, *P* = 0.002) (Table 3). As more robust markers for the outcome are particularly relevant in patients with a preoperative low-grade histology, we evaluated the prognostic value of MSH6 in curettage tissue with endometrioid grade 1–2 subtype. High expression of MSH6 predicted reduced survival with a 5-year DSS of 83%, compared to 94% for patients with low MSH6 expression (*P* < 0.001) (Fig. 3a). In multivariate cox analysis, MSH6 demonstrated independent prognostic impact within the low-grade patient group, after adjusting for age and hormone receptor status, with a multivariate HR of 2.9 (95% CI 1.6–5.4, *P* < 0.001) (Table 4).

**Table 3:** Prediction of poor disease-specific survival based on age, curettage histology, ER/PR and MMR protein expression in curettage specimens in endometrial cancer patients.

Risk factor	*N*	Univariate HR (95% CI)	*P*	Multivariate HR (95% CI)	*P*
Age	394	1.1 (1.1–1.1)	**<0.001**	1.0 (1.02–1.1)	**<0.001**
Curettage histology^a^					
Low grade	324	1		1	
High grade	70	8.5 (5.1–14.1)	**<0.001**	3.6 (1.97–6.7)	**<0.001**
ER/PR expression^b^					
High	335	1		1	
Low	59	5.7 (3.4–9.4)	**<0.001**	1.9 (1.02–3.4)	**<0.001**
MSH6					
Low	206	1		1	
High	188	4.5 (2.5–8.1)	**<0.001**	2.8 (1.46–5.3)	**0.002**
MSH2					
Low	136	1		1	
High	258	1.9 (1.1–3.5)	**0.020**	1.1 (0.61–2.1)	0.694
PMS2					
Low	221	1		1	
High	173	1.9 (1.2–3.1)	**0.009**	1.2 (0.7–2.1)	0.512

*MSH6* MutS Homolog 6, *MSH2* MutS Homolog 2, *PMS2* PMS2 Homolog 2.

Statistically significant *P*-values (<0.05) are bold.

^a^Curettage histological classification, low-grade (endometrioid grade 1 or 2) or high-grade (endometrioid grade 3 or non-endometrioid).

^b^Low ER/PR expression, loss of or low expression of ER and loss of PR expression.

Events: 64; Global *P* value (log-rank): <0.001.

**Table 4:** Prediction of poor disease-specific survival based on age, ER/PR and MSH6 protein expression in *low-grade* curettage specimens in endometrial cancer patients.

Risk factor	*N*	Univariate HR (95% CI)	*P*	Multivariate HR (95% CI)	*P*
Age	432	1.1 (1.1–1.1)	**<0.001**	1.1 (1.0–1.1)	**<0.001**
ER/PR expression^a^					
High	388	1		1	
Low	44	4.7 (2.6–9.0)	**<0.001**	4.4 (2.3–8.4)	**<0.001**
MSH6					
Low	264	1		1	
High	168	3.1 (1.7–5.7)	**<0.001**	2.9 (1.6–5.4)	**<0.001**

*MSH6* MutS Homolog 6.

Statistically significant *P*-values (<0.05) are bold.

^a^Low ER/PR expression, loss of or low expression of ER and loss of PR expression.

Events; 46; Global *P* value (log-rank): <0.001.

Given the strong prognostic potential of MSH6, a series of whole curettage sections (*n* = 106) overlapping with TMA cases was stained and scored using the same protocol. A substantial agreement in scoring results were seen between TMAs and whole sections (Cohen’s κ = 0.67, *P* < 0.001). In line with data from TMA scorings, high MSH6 evaluated on whole sections significantly associated with poor DSS (*P* = 0.013) (Fig. 3b).

### High MSH6 associates with copy number high tumours and predicts poor outcome within the MMR-D subgroup

**Fig. 4:**
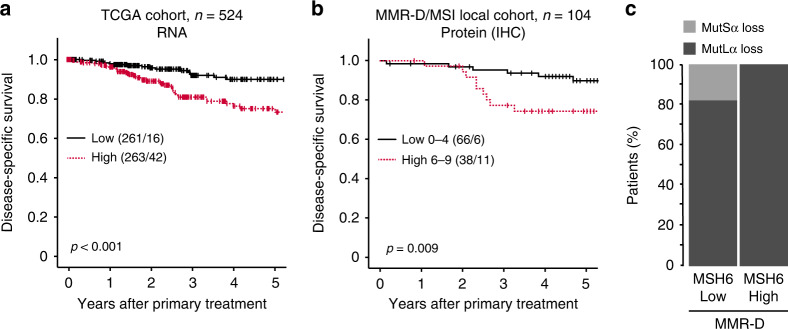
High expression of MSH6 is prognostic in the TCGA cohort and may risk-stratify patients with MMR-D tumours. In the TCGA cohort, high (mRNA > median) MSH6 associated with poor disease-specific survival (**a**). In the local cohort, high IHC expression of MSH6 associated with poor outcome in patients with mismatch repair deficient (MMR-D) tumours (**b**). Patients with high MSH6 expression have loss of the MutLα complex (**c**). TCGA The Cancer Genome Atlas, MSH6 MutS Homolog 6, MSI microsatellite instable.

The TCGA endometrial cancer (PanCancer Atlas) gene expression dataset was used to investigate the prognostic value of MSH6 in independent datasets. Mirroring results from protein expression, high *MSH6* associated with high-grade endometrioid and non-endometrioid types (*P* < 0.001 for both) (Table 5). High *MSH6* further associated with the TCGA molecular subgroup of copy number high tumours (*P* < 0.001) and predicted reduced survival with a 5-year DSS of 84% in the high expression group and 94% in the low expression group (*P* < 0.001, Fig. 4a).

**Table 5:** High *MSH6* mRNA expression associates with histological subtype and molecular subgroup in the TCGA cohort.

Variable	Low, *n* (%)	High, *n* (%)	*P* value
Histological subtype			**<0.001**
Endometrioid	239 (60)	158 (40)	
Serous	18 (17)	91 (83)	
Mixed	6 (29)	15 (71)	
Histological grade*			**<0.001**
Grades 1–2	164 (77)	49 (23)	
Grade 3	75 (41)	109 (59)	
Molecular subclass			**<0.001**
POLE	26 (53)	23 (47)	
MSI	92 (62)	56 (38)	
CN low	109 (74)	38 (26)	
CN high	27 (17)	136 (83)	

*POLE* DNA polymerase epsilon, *MSI* microsatellite instable, *CN* copy number.

Statistically significant *P*-values (<0.05) are bold.

*Only endometrioid histology.

Data missing on the histological subtype for 2 patients, the histological grade for 2 patients, and molecular subclass for 22 patients.

Interestingly, in our local cohort, within MMR-D cancers, we found high MSH6 protein expression in 38/104 patients. This subgroup had a significantly worse outcome than MMR-D patients with low MSH6 expression (5-year DSS of 71% and 91%, respectively; *P* = 0.009) (Fig. 4b). The high MSH6 expression group had loss of MLH1/PMS2 (MutLα), whereas the MSH6 low expression group had loss of either MLH1/PMS2 (MutLα) or MutSα (Fig. 4c).

## Discussion

Immunohistochemical staining of MMR proteins is entering the preoperative workup as part of the molecular classification of endometrial tumours [4]. Identification of patients with MMR-D tumours provides prognostic information and helps stratify patients for treatment with immune checkpoint inhibitors. Here we report a substantial agreement in MMR detection between preoperative and operative samples and demonstrate the added value of identifying patients with high MSH6 expression, a subgroup associated with more aggressive disease and significantly worse outcome.

About 20–30% of endometrial cancer patients have tumours with deficient MMR [5, 12, 31–33]. MMR deficiency is largely determined from hysterectomy samples in research settings, while in the clinical workup, preoperative biopsies are used for diagnostics. Previous reports only evaluated MMR status agreement in 14 and 15 patients, respectively [12, 32]. This is, to our knowledge, the first study that uses a large prospectively collected population-based endometrial cancer cohort to evaluate MMR status agreement between paired preoperative and operative samples. We identify a substantial agreement between paired samples, supporting the use of preoperative biopsy for MMR status detection in endometrial cancer. Still, it should be noted that MMR status was discordant in 7.6% of our paired samples. Tumours with subclonal loss of mismatch repair protein(s), or loss in only one available sample, are considered MMR-D [26]. Among the tumours detected as MMR-D in our study cohort, 17% (22/130) were defined as MMR-D only after evaluating the hysterectomy sample. Discrepancies may be due to methodological issues but is more likely to reflect subclonal MMR protein expression. This is commonly observed in a subset of endometrial tumours [26] and reported to occur in 7.2% of samples [34]. Thus, in a small fraction of MMR-D patients, the tumours may be falsely classified as MMR-P using the preoperative biopsy. These tumours are, except for rare cases, *POLE* and *TP53* wild-type [5, 30] and thus allocated (incorrectly) to the copy number low subgroup. According to the ESGO/ESTRO/ESP guidelines, this will not affect the primary treatment of patients [4]. However, in the recurrent setting, re-evaluation of MMR status in the operative biopsy should be considered as this may qualify for treatment with immune checkpoint inhibitor.

Studies have previously suggested MSH6 as a potential prognostic marker in endometrial cancer, where high MSH6 in hysterectomy tissue is associated with poor outcome and non-endometrioid subtype [19, 35]. Our data validate these findings and demonstrates a strong prognostic value in preoperative samples in this large prospective endometrial cancer cohort. Prognostic value of MSH6 was further validated in hysterectomy tissue, at both protein and mRNA levels, and in the external TCGA endometrial cancer cohort. In addition, within the subgroup of patients with endometrioid low-grade histology, we identify MSH6 as an independent predictor of poor survival. According to most guidelines, these patients are not offered more invasive surgery nor adjuvant therapy, overlooking a small subset of patients (at least 7%) [36] that has increased likelihood of disease recurrence. Furthermore, MSH6 associated with prognosis within the MMR-D subgroup, i.e., patients with loss of the MutLα complex combined with high MSH6 expression had poorer survival (DSS = 71%) than the remaining MMR-D patients with low or loss of MSH6 expression (DSS > 90%), similar to that reported for the POLE subgroup [5, 12, 37]. These patients are considered to have very low risk with no need for adjuvant treatment. Collectively, if MSH6 is thoroughly evaluated, this marker may aid in prognostication of endometrial cancer patients preoperatively, thus refining patient stratification for invasive surgery and adjuvant therapy, and in addition function as an MMR-D classifier [12, 31]. Also, the added benefit of MSH6 intensity scoring argues for the use of IHC over MSI assay for MSI subgroup classification, which is supported by the recent approval of VENTANA MMR RxDx Panel (Roche) for IHC MMR-D detection in solid tumours.

High MSH6 also yields prognostic value across other cancer types, suggesting that MSH6 may function to promote aggressive tumour behaviour [13–17, 19]. However, the mechanism underlying overexpression of MSH6 is largely unknown. Upregulation may be induced by higher proliferation rates to ensure sufficient repair of mismatches [38], but studies investigating its function suggest that upregulation of MSH6 or other MMR proteins induces genomic instability [39, 40]. Interestingly, we found an enrichment of high MSH6 tumours in the CN-high subgroup. MMR-induced genomic instability may promote tumour progression by accelerating tumour evolution and thus accumulating more aggressive subclones. However, it is proposed in glioblastoma that high MSH6 promotes aggressive cell phenotypes more directly by acting through a MSH6-CXCR4-TGFβ1 feedback loop that regulates p-STAT3/Slug and p-Smad2/3/ZEB2 signalling pathways [41]. Collectively, these studies suggest high MSH6 as a driver of aggressive disease, which should also be investigated in endometrial cancer.

In conclusion, we here demonstrate that MSH6 can be used preoperatively as an independent prognostic marker in addition to its value as MMR-D classifier in endometrial cancer. This information can be provided at low costs and may be important for treatment decisions. However, additional studies and in particular prospective randomised trials would be important to validate the prognostic value and effect of implementing preoperative MSH6 scoring in clinical routine.

## Supplementary information

Reporting summary

Supplementary Material

## Data Availability

Not applicable.
