# Supplementary material for: Mismatch repair markers in preoperative and operative endometrial cancer samples; expression concordance and prognostic value
Source: Br J Cancer. 2022 Dec 8;128(4):647–55. doi: 10.1038/s41416-022-02063-3 (PMC9938259; doi:10.1038/s41416-022-02063-3)
Supplement: Supplementary file 2 — Supplementary Material [file 41416_2022_2063_MOESM2_ESM.pdf]

Supplementary Table 1. MMR protein expression levels associates with clinicopathological variables in hysterectomy specimens

Variable	MSH6 Low n (%)	MSH6 High, n (%)	P-value	MSH2 Low n (%)	MSH2 High n (%)	P-value	PMS2 Low n (%)	PMS2 High n (%)	P-value	MLH1 Low n (%)	MLH1 High n (%)	P-value
**Number of patients**	380	332		502	261		265	236		346	357	
**Age**			**0.001**			**0.006**			**0.039**			0.894
< 66	214 (60)	145 (40)		278 (70)	117 (30)		148 (57)	110 (43)		182 (50)	186 (50)	
≥ 66	166 (47)	187 (53)		224 (61)	144 (39)		117 (48)	126 (52)		164 (49)	171 (51)	
**Histologic type***			**< 0.001**			**< 0.001**			**< 0.001**			**< 0.001**
Endometrioid	335 (58)	239 (42)		434 (71)	177 (29)		220 (59)	156 (41)		296 (53)	264 (47)	
Non-endometrioid	45 (33)	93 (67)		68 (45)	84 (55)		45 (36)	80 (64)		50 (35)	93 (65)	
Clear cell	17 (63)	10 (37)		22 (76)	7 (24)		4 (21)	15 (79)		12 (44)	15 (56)	
Serous papillary	17 (27)	47 (73)		28 (38)	45 (62)		25 (38)	41 (62)		23 (34)	45 (66)	
Carcinosarcoma	8 (24)	26 (77)		10 (29)	25 (71)		11 (39)	17 (61)		9 (27)	24 (73)	
Undifferentiated/other	3 (23)	10 (77)		8 (53)	7 (47)		5 (42)	7 (58)		6 (40)	9 (60)	
**Histologic grade****			**0.016**			**0.038**			0.662			0.781
Grade 1-2	285 (61)	181 (39)		361 (73)	132 (27)		174 (59)	121 (41)		240 (53)	212 (47)	
Grade 3	47 (48)	51 (52)		67 (63)	39 (37)		41 (56)	32 (44)		50 (51)	47 (49)	
**Myometrial infiltration**			0.531			0.648			0.909			0.066
< 50%	238 (54)	202 (46)		307 (67)	154 (33)		169 (53)	148 (47)		207 (47)	238 (54)	
≥ 50%	139 (52)	130 (48)		193 (65)	104 (35)		95 (53)	85 (47)		137 (54)	118 (46)	
**FIGO stage**			**0.001**			**0.039**			0.480			0.861
I-II	334 (56)	262 (44)		431 (67)	209 (33)		223 (54)	193 (46)		293 (49)	304 (51)	
III-IV	46 (40)	70 (60)		71 (58)	52 (42)		42 (49)	43 (51)		53 (50)	53 (50)	
**Ploidy**			**< 0.001**			**< 0.001**			**0.011**			0.071
Diploid	191 (56)	151 (44)		257 (68)	124 (33)		139 (57)	105 (43)		179 (52)	166 (48)	
Aneuploid	33 (32)	69 (68)		52 (46)	60 (54)		32 (40)	47 (60)		43 (42)	60 (58)	
**Metastatic lymph nodes**			**0.022**			**0.003**			0.901			0.349
Negative	273 (54)	228 (46)		373 (69)	167 (31)		189 (54)	160 (46)		245 (49)	259 (51)	
Positive	27 (40)	41 (60)		36 (51)	34 (49)		27 (55)	22 (45)		33 (55)	27 (45)	

FIGO: International Federation of Gynaecologist and Obstretics, n: number of patients, MSH6: MutS homolog 6, MSH2: MutS homolog 2, PMS2: PMS1 homolog 2, MLH1: MutL homolog 1
MSH6, MSH2, MLH1; Low: SI 0-4, high: SI 6-9, PMS2; Low: SI 0-3, high: SI 4-9.
*Comparing endometrioid vs. non-endometrioid
**Only endometrioid histology
Data missing on histologic grade for 10 (MSH6), 12 (MSH2), 8 (PMS2), and 11 (MLH1) patients, myometrial infiltration for 3 (MSH6), 11 (MSH2), 5 (PMS2), and 3 (MLH1) patients, ploidy for 268 (MSH6), 270 (MSH2), 323 (PMS2), and 255 (MLH1) patients, metastatic lymph nodes for 143 (MSH6), 153 (MSH2), 103 (PMS2), and 139 (MLH1) patients.

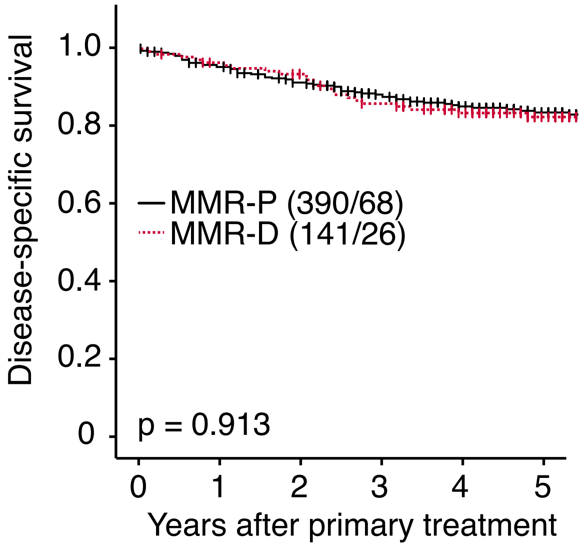

Supplementary Figure 1. No difference in survival outcomes between patients with MMR-P and MMR-D status in curettage samples. MMR-D: mismatch repair deficient, defined by loss of either MSH6, MSH2, PMS2 or MLH1. MMR-P: mismatch repair proficient. P-values are given by log-rank (Mantel-Cox) analysis. Number in brackets indicates total number of patients/number of events.

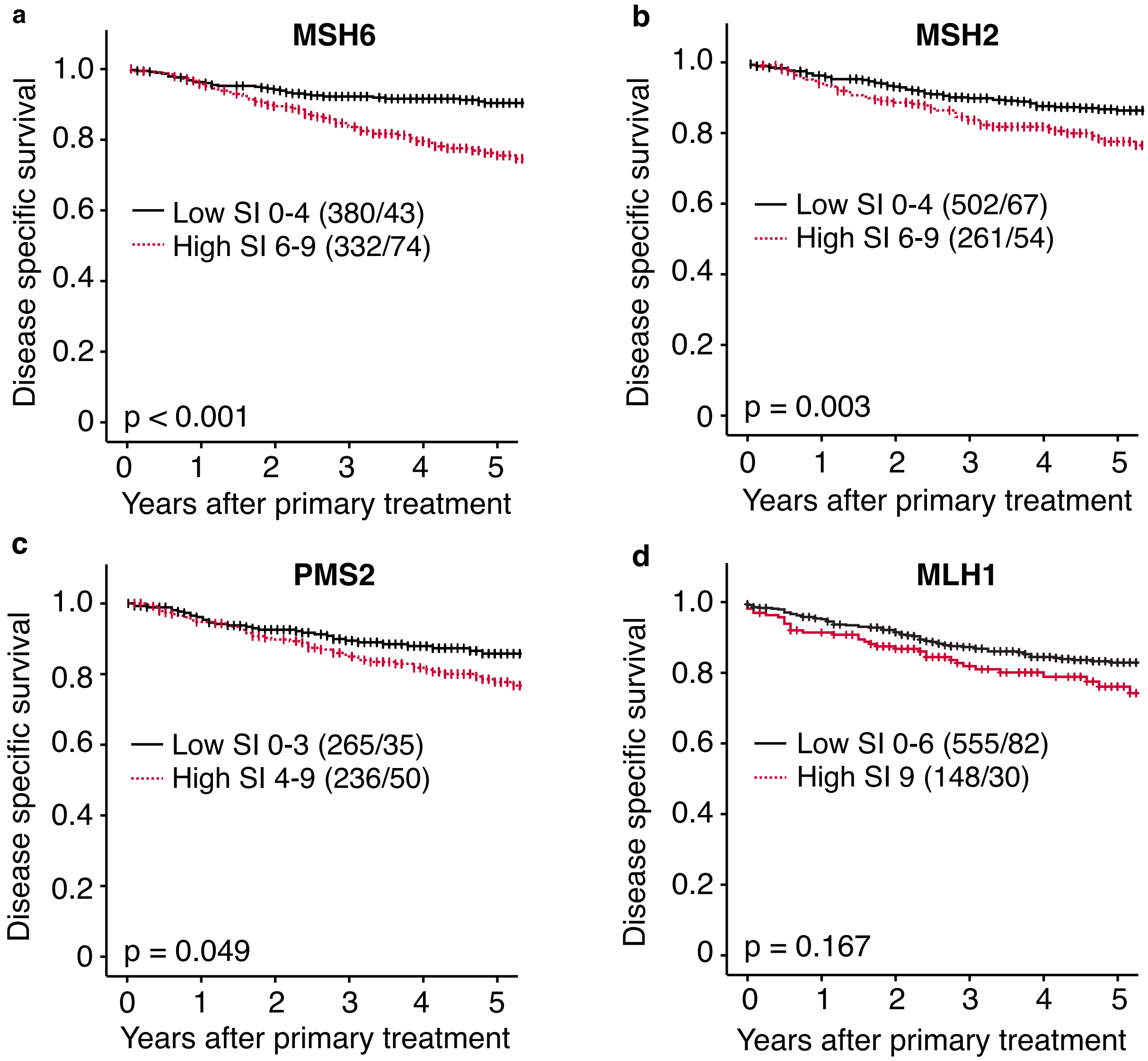

Supplementary Figure 2. Expression of MSH6, MSH2 and PMS2 is prognostic in hysterectomy specimen. High expression of MSH6 (a), MSH2 (b) or PMS2 (c) in hysterectomy was associated with poor outcome in endometrial cancer. MLH1 expression (d) was not associated with survival. P-values are given by log-rank (Mantel-Cox) analysis. Number in brackets indicates total number of patients/number of events. Abbreviations: MutS Homolog 6 (MSH6), MutS Homolog 2 (MSH2), PMS2 Homolog 2 (PMS2), MutL Homolog 1 (MLH1).

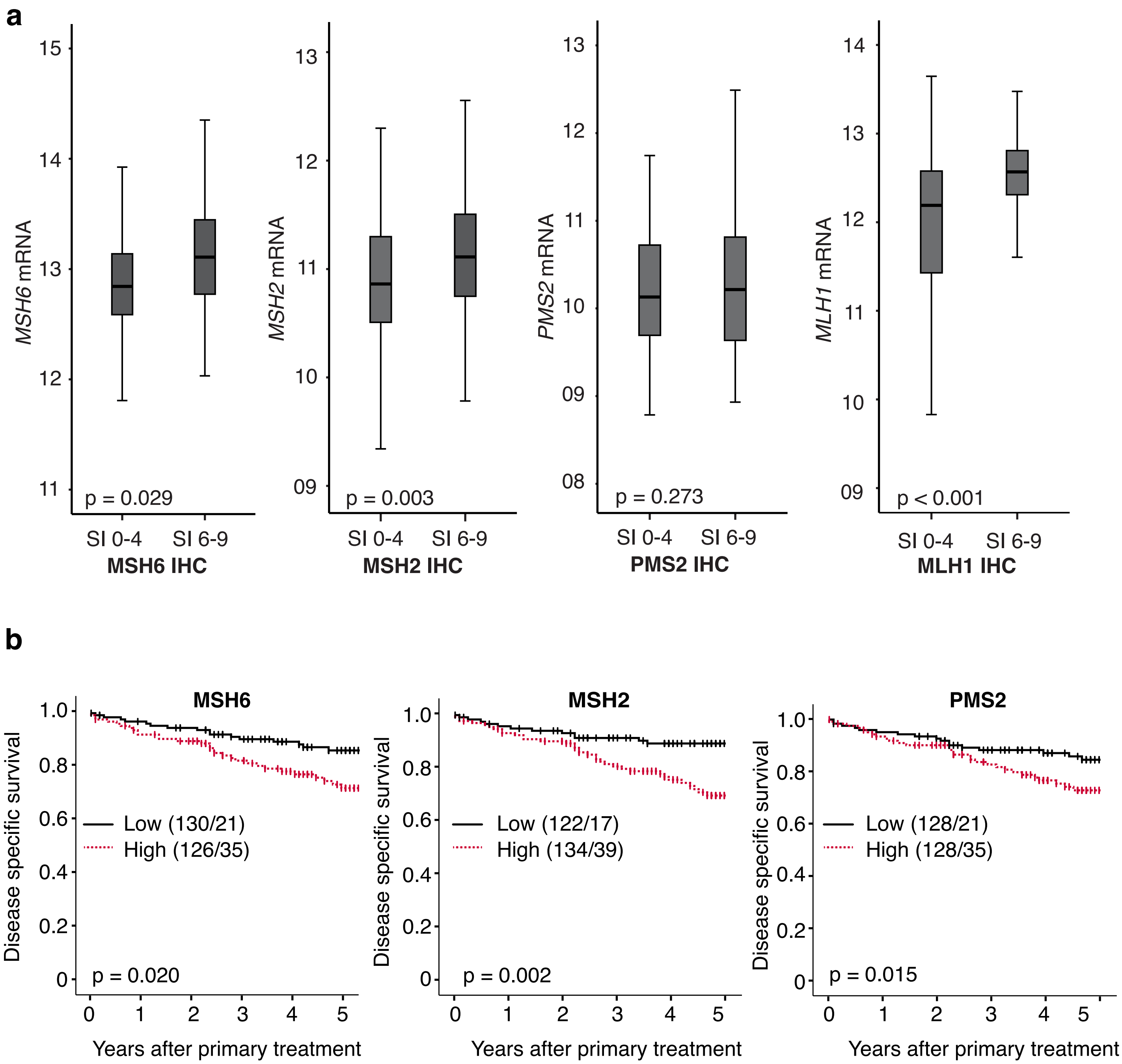

Supplementary Figure 3. MSH6, MSH2 and PMS2 mRNA expression is prognostic in hysterectomy specimen. *MSH6*, *MSH2*, *MLH1* or *PMS2* (median) mRNA expression in relation to corresponding immunohistochemical protein levels (median) in hysterectomy tissue (a). mRNA expression of MSH6, MSH2 and PMS2 associated with disease-specific survival in hysterectomy samples (b). Median was used as cut-off for low and high expression groups. P-values are given by log-rank (Mantel-Cox) analysis. Number in brackets indicates total number of patients/number of events. Abbreviations: MutS Homolog 6 (MSH6), MutS Homolog 2 (MSH2), PMS2 Homolog 2 (PMS2).
